# Technological advances: Have they improved standards? Review of outcomes from the Welsh cervical screening programme

**DOI:** 10.1177/0969141320918270

**Published:** 2020-04-16

**Authors:** Alejandra Castanon

**Affiliations:** King’s College London, Faculty of Life Sciences & Medicine, School of Cancer & Pharmaceutical Sciences, Cancer Prevention Group, London, UK

**Keywords:** Cervical Screening Wales, Performance Indicators, role of technology, liquid-based cytology, HPV testing

## Abstract

**Objectives:**

Introduction of new technologies into cervical screening programmes has allowed more efficient programmes with less resources. We present an overview of screening technologies introduced into the Cervical Screening Wales programme and their evolution over time.

**Methods:**

Data from the programme’s statistical report were used to evaluate its performance over a 17-year period between 2001/02 and 2017/18.

**Results:**

The introduction of liquid-based cytology has had a substantial impact on reducing inadequate sample rates and on increasing the positive predictive value of cytology. Inadequate rates have increased following the implementation of human papilloma virus testing as a triage test for cytology. Further knock-on effects on standard reporting ranges are expected following the introduction of human papilloma virus testing as the primary screening test. New performance standards have been introduced to better reflect the performance of the programme at a time when disease prevalence is expected to fall as women vaccinated against human papilloma virus reach screening age.

**Conclusions:**

Improvements to this cervical cancer screening programme as illustrated through performance indicator ranges suggest a major role played by technology.

## Background

The Pap test (also known as the smear or cytology test) was developed by George Papanicolaou in the early 1940s.^1^ Health authorities in the United Kingdom began offering cervical screening in the 1960s as a test to detect precancerous cervical cells (cervical intraepithelial neoplasia, CIN) with the aim of treating them to prevent cancer. By 1967, cervical screening had been introduced across the whole UK National Health Service (NHS). It took two more decades before evidence that screening reduced the incidence of and mortality from cervical cancer became irrefutable.^2–4^ In 1988, the UK introduced a computerised call/recall system and recommended that women aged 20 to 64 be screened every three to five years.^5,6^

Although call/recall was introduced in Wales from 1988, a unified screening programme did not come into force until 1999 when Cervical Screening Wales (CSW) was launched by Welsh Health Minister, Jon Owen Jones.^7^ CSW would be responsible for the cervical screening programme, including management and co-ordination, call and recall arrangements, cytology sample taking, cytology services, histology services and colposcopy services.^8–10^

All organised screening programmes have quality measures to assess and maintain high standards.^11,12^ These are called performance standards. Given that cervical cytology was introduced into routine practice without evidence from randomized controlled trials, there is no ‘gold standard’ against which performance can be compared. This makes setting performance standard indicators for cervical screening particularly difficult.

Since 1998 there have been several technological advances introduced by CSW. This review uses data from the Welsh cervical screening programme to evaluate its performance over a 17-year period between 2000 and 2017. The focus is on four performance indicators: inadequate sample rates, proportion of adequate tests reported as high-grade dyskaryosis, sensitivity to high-grade disease, and the positive predictive value (PPV) of high-grade dyskaryosis for histologically confirmed CIN grade 2 or worse disease. The aim is to present a qualitative description of the evolving programme and a quantitative audit of trends in standard reporting ranges over a 17-year period between 2001/02 and 2017/18.

## Performance standards in cervical screening

Although the launch of the organised screening programme in 1988 drew attention to quality issues, training requirements and the importance of early and effective treatment, it was not until the publication in 1995 of the document “**A**chievable standards, **B**enchmarks for reporting and **C**riteria for evaluating cervical cytopathology” (also known as the ABC Report) by the English NHS Cervical Screening Programme^13^ that standard ranges for cervical cytology were established. In fact, before 1995, there were few explicit performance standards for cytology sample takers and no coordinated system for monitoring performance.

Defining the optimal range for cervical screening performance standards is challenging as it depends on the relative value given to a high specificity or a high sensitivity. Performance ranges in the first ABC Report were derived from a survey of two consecutive years’ results from 12 selected laboratories in England. In the second ABC Report, the standards were reviewed as more years of data become available, but performance ranges were still based on reference ranges from laboratories in England. Hence, performance indicators concentrate on identifying outliers in the data and subjecting them to scrutiny, ensuring a more even service across the country and identifying those laboratories where further investigation may be required. These indicators were adopted by CSW from 1999 onwards.

Performance indicators were updated in the latest version of the ABC (3rd version)^14^ document to reflect changes to the national programme, including the use of liquid-based cytology and the implementation of human papilloma virus (HPV) triage. The new standards were designed to produce a more uniform national programme, enabling it to operate at an optimal ratio of maximum benefit to minimum harm.^15^ The Welsh programme began reporting these new values from 2014/15 onwards.

A summary of performance indicators and how these have changed over time can be found in Table 1. This review focuses on the following indicators:

**Table 1.** Criteria for evaluating cervical cytology in the UK.

Performance standard	ABC1 (1995)^13^	ABC2 (2000)^41^	ABC3 (2013)^14^
	Pre-specified percentiles within which laboratory values should fall		
Laboratory cytology test results^a^	10th–90th percentile	10th–90th percentile	5th–95th percentile
Moderate and severe (% adequate)	1.2%–2.0%	1.0%–2.0%	No longer mandatory
Mild and borderline (% adequate)	4.0%–7.0%	4.1%–9.5%	No longer mandatory
Inadequate (% all tests)	5.0%–9.0%	5.8%–12.9%	To be estimated yearly
Sensitivity of primary screening^b^ for moderate or worse^c^ For high grade	85–95%	≥90% ≥95%	No longer mandatory
Positive predictive value (PPV) of moderate/severe dyskaryosis for CIN2 or worse^b^	65–85%	65–90%	To be estimated yearly
Referral value (RV): # of women referred to colposcopy to detect one CIN2+	NA	NA	Estimated yearly
Mean CIN score (MCS)^d^	NA	NA	Between 1.5 and 2.5
Abnormal predictive value (APV): calculates the percentage of samples reported as borderline or low grade that led to referral and subsequent diagnosis of CIN2 or worse	NA	NA	Estimated yearly
HR-HPV-positive rate for borderline/low-grade samples: an analysis of variation in the HR-HPV-positive rate	NA	NA	This is an optional performance measure.

a Duplicate test results following colposcopy must be excluded from the figures for moderate and severe dyskaryosis.

b Performance indicator ranges for sensitivity of primary screening based on rapid review.

c Achievable standards for the sensitivity of primary screening, for a final report of moderate dyskaryosis or worse (ABC2 changes it to all abnormalities) to be calculated in respect of the final report issued by the laboratory following rapid review. For PPV calculated as the % of women referred with moderate or worse whose biopsy is reported as CIN2+ (ABC3 changes this to high grade or worse).

d The mean CIN score (MCS) is a method to summarise the distribution of detected CIN cases as a single figure. It is based on all adequate cytology samples with adequate histology outcomes and is an optimal performance measure. A high value indicates that more women referred to colposcopy were diagnosed with high-grade CIN.

- proportion of adequate tests reported as inadequate,
- proportion of adequate tests reported as moderate or worse dyskaryosis,
- positive predictive value (PPV) – proportion of women referred with moderately/severe dyskaryosis whose biopsy is reported as CIN2+, and
- sensitivity of primary screening – proportion of women with a CIN2+ who had a cytology result of moderate/severe dyskaryosis.

The four new performance standard measures introduced in ABC3 are:

- Referral value (RV) – number of women referred to colposcopy to detect one CIN2+.
- Mean cervical intraepithelial neoplasia score (MCS) – a method to summarise the distribution of detected CIN cases as a single figure.
- Abnormal predictive value (APV) – calculates the percentage of samples reported as borderline or low grade that led to referral and subsequent diagnosis of CIN2 or worse.
- High-risk (HR) HPV-positive rate for borderline/low-grade samples.

## Technological advances introduced into CSW between 1999 and 2016

### Rapid re-screening

“Rapid re-screening” was first proposed as a method for routine quality control of primary screening (using cytology) in 1995. Rapid re-screening of all tests was recommended over selective double screening of 10% of negative samples at normal speed because it was shown to be as sensitive in preventing false negatives.^13,16^ It was therefore recommended that all slides considered negative or inadequate be subject to a rapid (60 s) re-screen.^16^

At the time of the introduction of the Welsh screening programme (in 1999), all laboratories in Wales operated a system of rapid review of all slides declared normal or inadequate at initial screen (in England 96% of laboratories used the technique at the time). However, the time spent reviewing each slide varied between laboratories, and at least two laboratories seeded known positive slides into those to be rapid reviewed as a test of the effectiveness of the process.^8^ CSW standardised rapid review across laboratories and embedded it into good practice.

### Liquid-based cytology

Liquid-based cytology (where the cells are deposited into a small pot containing preservative fluid before being transferred onto a glass slide at the laboratory) has been favoured over conventional cytology because it leads to lower rates of inadequate samples.^17^ Additionally, evidence suggests that rapid pre-screening is more sensitive in LBC than in conventional cytology.^18^ To date, there are two major liquid-based cytology platforms: ThinPrep® (Hologic) and SurePath™ (Becton Dickinson). Both technologies have the necessary regulatory approvals to be used within UK screening programmes but they use different preservative fluids and employ different methodologies in sample preparation, so laboratories must choose one.

In Wales, a pilot across four laboratories was set up in 2001 using ThinPrep® to assess whether LBC should be implemented fully across Wales. The pilot was successful^19^ and a full roll-out of the technology was authorised in February 2004 with a concomitant training programme for laboratory staff and cytology sample takers which was completed in June 2005. However, CSW decided that, for the full conversion, SurePath™ technology would be adopted nationally. The last laboratory to convert to LBC did so by January 2006. Since then all cervical smears taken in Wales have been processed using LBC technology.^10^

### No further review

Computer-assisted technology (using BD FocalPoint™ Slide Profiler) became operational in Wales in October 2012.^20^ The system analyses images to detect squamous carcinoma and adenocarcinoma, and their precursor lesions, in cervical cytology preparations.^21^ The main benefit is workload reduction. A manual quality assurance check prior to a negative report being issued is only required for about 20% of cervical cytology samples identified as negative by the technology.

Once HPV primary testing is fully rolled out and the cytology workload is substantially reduced, rapid review and no further review technology may become obsolete.

### HR-HPV testing

High-risk HPV, a common sexually transmitted infection, is the underlying cause of cervical cancer.^22^ At least 13 HPV sub-types have been identified as high risk for causing cancer. Around 70% of cervical cancers are caused by just two types, HPV 16 and 18.^23^

There is strong evidence that testing for the presence of HR-HPV virus is more effective at protecting against invasive cervical cancer than cervical cytology.^24,25^ The European Union as of 2015 recommended that HPV-based screening should be the preferred primary screening method for women aged over 30 years.^26^

HR-HPV testing was introduced in September 2014 to Wales as triage for borderline and mild cytology. However, full implementation did not occur until May 2016. Following a full economic evaluation^27^ and a pilot study involving 20% of the population in Wales, as of September 2018, HPV primary testing was introduced into the Welsh screening programme for women aged 25–64 years.

### Vaccination against HR-HPV types

Vaccination against HPV types 16 and 18, with the bivalent (Cervarix) vaccine, was rolled out nationally in 2008 to girls aged 12–13 with a catch-up programme for those aged 15 to 18.^28^ In September 2015, the first vaccinated catch-up cohorts reached the age of 25 and became eligible for screening. The first cohort of women vaccinated at ages 12–13 will enter screening in 2020. Since September 2011, the quadrivalent vaccine (Gardasil) has been used.

### Screening intervals and age ranges

From 1999 (until September 2013), screening was offered to women aged 20–64 at a three-yearly recall interval. In 2013, the Welsh Government announced that, in line with recommendations made by the UK National Screening Committee, the age range covered by Cervical Screening Wales and the frequency of invitation would change in response to evidence that screening women under the age of 25 is not effective at preventing cervical cancer, and the lack of evidence to suggest that women over the age of 50 need to be screened more frequently than once every five years. From the 1st of September 2013, women born after 1 September 1993 have been invited for cytology tests at age 25 and any woman aged between 50 and 64 who attends screening is re-invited every five years rather than every three years.^29^ This means that a woman screened at age 60 years will now be exited, whereas previously she would have been invited again at age 63. As of April 2019, there were no plans to extend the screening intervals in women receiving HPV primary testing in Wales.^30^

## Impact of increased quality assurance and new technologies on the Welsh screening programme

### Impact on laboratory organisation

In 1999, there was considerable variation in both organisation and performance of the screening programme among cytology and histology laboratories. At the time, cervical cytology from Wales was processed and read at 13 laboratories in Wales and 3 in England. Only 8 out of the 13 in Wales had full and unconditional accreditation by the Clinical Pathology Accreditation (CPA) body.

Following the launch of CSW, standards were set out to which all cytology and histology laboratories should aim to be performing.^8^ These standards mandated that all laboratories be fully CPA accredited, all terminology for reporting should be British Society for Clinical Cytology (BSCC) terminology,^31^ and that laboratories be monitored against standard reporting ranges. To ensure standards were maintained, the minimum number of hours staff could work without a break and number of slides that staff needed to review per year were also specified.

Standardisation of laboratory practices, including standardisation of rapid re-screening, allowed for the 13 original laboratories in Wales when CSW was formed to be consolidated into 11 laboratories by 2001. A further reorganisation of pathology services resulted in the 11 laboratories being consolidated into seven in 2011/12. A year later, the services consolidated further into four laboratories.^32^ Given that reporting of HPV results can be automated and will lead to a significant fall in cytology workload, the four remaining laboratories in Wales were consolidated into one (at Magden Park) in September 2018.^30,33^

### Impact on performance standards

**Figure 1.**
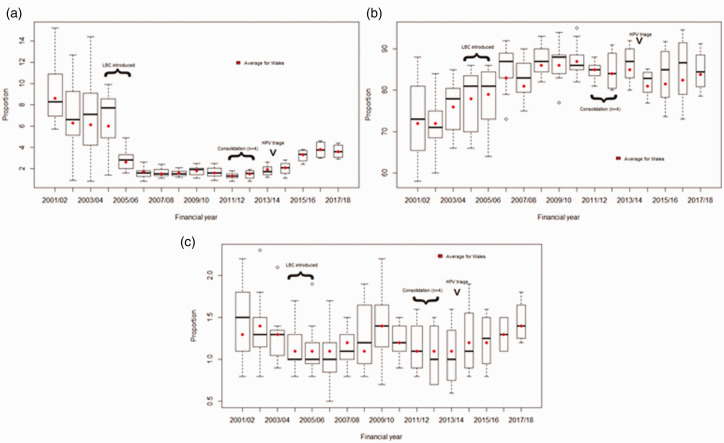
Laboratory performance indicators for Wales between 2001/02 and 2017/18 financial years (based on one observation per laboratory per year). (a) Proportion of all cytology tests reported as inadequate. (b) PPV of high-grade dyskaryosis for CIN2 or worse. (c) Proportion of adequate tests reported as moderate or severe dyskaryosis. Boxes show the 75th percentile and 25th percentile and the line in the middle shows the median (of the distribution across laboratories). A closed circle shows the average for Wales. Dotted bars indicate the range except when there are outlying laboratories whose values are indicated by open circles. There were 11 laboratories in Wales between 2001 and 2010, seven in 2011, and four from 2012 onwards.

**Figure 2. fig2-0969141320918270:**
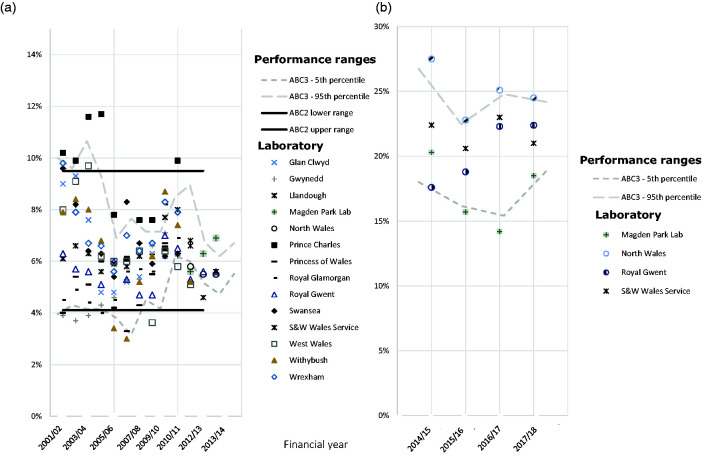
Performance indicators for Wales between 2001/02 and 2017/18 financial years. (a) Proportion of all cytology tests reported as borderline or mild between 2001/02 and 2013/14. (b) APV between 2014/15 and 2017/18. Dashed lines indicate the 5th to 95th percentile range; solid lines indicate ABC3 performance standard ranges.

Data from the annual statistical returns (known as KC61 returns) are used to evaluate performance standards for the CSW programme.^15^ These data are summarised yearly in statistical reports publicly available for the 2001/02 financial year onwards. To illustrate the effect of quality assurance and new technologies on performance standards, data from the CSW statistical reports are summarised graphically for a 17-year period between the financial years 2001/02 and 2017/18 in Figures 1 [Fig fig2-0969141320918270]to 3.^34^ The data for 2018/19 include results separately for four laboratories (consolidation did not occur until October 2018 and statistical reports are over financial year). However, given that this was a key transition year for the programme, the results are mentioned in the text although not displayed in the figures.

The impact of implementing liquid-based cytology is best observed in the proportion of inadequate tests reported over time in Wales. The overall inadequate rate in Wales in 2001/02 was 8.6%. From 2004/05, when the roll-out of LBC was initiated, the inadequate rate fell to 2.6% and by 2006/07 when the roll-out was completed, the rate dropped to under 2% (Figure 1(a)).

Since the roll-out of HPV triage, cytology inadequate rates in Wales have slowly been increasing to an average of 3.6% in 2017/18 (Figure 1(a)). The consolidation of laboratories and the preparations for the implementation of HPV primary screening have resulted in staff shortages. The reporting times for cytology have exceeded the standard 14-day turn around. Further cytology is now carried out on the sample that is left after HPV testing has been done (whereas it used to be the other way around). This period of transition has probably played a role in the increase in cytology tests being reported as inadequate. It is noteworthy that a cytology inadequate rate of 4.5% is reported for Wales in 2018/19. However, the overall number of cytology tests within the programme has fallen so that they represent only 37.5% of all screening tests in Wales. Were one to take into account the HPV test in the calculation, the overall inadequate rate in Wales would fall to 1.8%.^34^ To put this in context, the 5th–95th percentile range of inadequate tests among 51 laboratories in England (2016/17) was 1.0–4.3% at a time when only 5% of the population were being tested with HPV as a primary test.^35,36^

A similar situation to that observed with the proportion of inadequate slides can be observed with the PPV of moderate or worse cytology for a diagnosis of CIN2 or worse (Figure 1(b)). With increased quality assurance and the introduction of LBC, the PPV across laboratories had been increasing and the spread between laboratories narrowing. This reached a peak in 2010/11 when no laboratory had a PPV below 80% and the national average remained around 85%; that is until 2012/13 when laboratories were consolidated. Since then the PPV has decreased and the spread between laboratories has increased; however, overall the PPV remains high (around 82%), Figure 1(b). The PPV in Wales is well within the range observed in England^36^ (76.7–92.3%) and in a recent systematic review of 27 studies where the PPV (of high-grade squamous intraepithelial lesions) was on average 77.5%.^37^

Simulation studies have shown that as cancer prevention programmes strengthen and the prevalence of disease decreases the PPV of cytology could decrease by as much as 50%,^38,39^ particularly once vaccinated women reach screening age. A recent Australian study has shown that decreases in the PPV of high-grade cytology were limited to cohorts of young women likely to have been vaccinated.^40^

**Figure 3.**
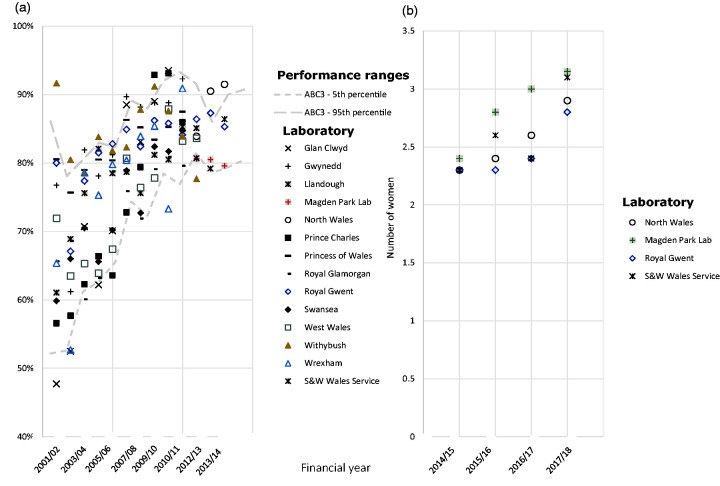
Performance indicators for Wales between 2001/02 and 2017/18 financial years. (a) Sensitivity of cytology to high-grade disease between 2001/02 and 2013/14. (b) RV between 2014/15 and 2017/18. Dashed lines indicate the 5th to 95th percentile range.

It has been well documented that some areas of the UK have a higher incidence of pre-cancerous disease than others.^15^ In preparation for further background changes in incidence of cervical dyskaryosis, reporting the proportion of low-grade slides and the sensitivity of cytology to high-grade has been replaced by the abnormal predictive value (APV) and the referral value (RV) (Table 1). To understand the value of the new performance standard measures, one only needs to consider that a report of 6% of all cytology tests being reported as low-grade dyskaryosis is much less informative than knowing that around 20% of all low-grade disease on cytology results in a high-grade (CIN2+) histological abnormality (Figure 2). Similarly, knowing that 3.0 women need to be referred to colposcopy to detect one CIN2 or worse provides better context than a report of 85% sensitivity of cytology to high-grade disease (Figure 3). For context in England (2016/17), the 5th to 95th percentile range for APV was 6.8–26.7% and the RF was 2.0–5.0.

In Wales, from 2001/02 to 2017/18, on average 1.3% of adequate tests were reported as moderate or severe dyskaryosis (Figure 1(c)).^34^ A peak is observed in 2008/09 and 2009/10 most likely related to the Jade Goody effect,^41^ which resulted in more women with a previous abnormality or who were lapsed attending screening. From 2014/15, when HPV triage was introduced, there has been a slight increase in the proportion of tests reported as moderate or severe dyskaryosis; however, this did not exceed 1.5% by 2017/18. The 2018/19 figures show that 2.6% of tests were reported as high-grade. This huge increase is expected when one considers that most of the cytology samples taken are triggered by a positive HPV test result. The proportion of tests reported as high-grade is likely to be further affected by the decreasing prevalence of vaccine-associated HPV types leaving less carcinogenic HPV types to be detected. There is also concern that the cytology workforce may become deskilled in an era when high-grade cytology becomes rare.

## Discussion

Performance indicators summarised here suggest a strong role of technology in improving the screening programme. However, there are several important considerations that limit the inferences that can be made from the data presented. The lack of a golden standard and the absence of randomised controlled trials make the evaluation of this screening programme particularly challenging. Data collected through the annual statistical returns (KC61) do not allow for stratification of performance standards by age or screening interval, both of which could account for the changes observed.

Continuous changes to the screening programme have made quality assurance difficult. In the coming years, performance indicators will be the first available statistics on which to evaluate the response of organised programmes to the rapidly changing times. Although the data presented here are specific to the Welsh screening programme, the impact of liquid-based cytology, HPV testing and vaccination on performance indicators is generalisable to other settings where organised screening is offered.

As has been illustrated here, cervical screening performance measures are susceptible to changes in technology, laboratory capacity and disease prevalence. Given that most screening programmes are now using HPV as the primary screening test and that vaccinated women are reaching screening age, over the coming years, careful monitoring of programme standards will be more important than ever. In addition, screening programmes should prepare mitigation strategies such as extending screening intervals, introducing new triage tests, and improving training and resilience of the workforce.
